# Median Nerve Passing below the Ulnar Head of Pronator Teres in Cadavers of a Medical College in Western Nepal: A Descriptive Cross-Sectional Study

**DOI:** 10.31729/jnma.7230

**Published:** 2022-01-31

**Authors:** Nitasha Sharma, Ruku Pandit, Sandip Subedi

**Affiliations:** 1 Department of Anatomy, Universal college of Medical Science and Teaching Hospital, Bhairahawa, Nepal; 2 Department of Anatomy, College of Medical Science and Teaching Hospital, Bharatpur, Nepal; 3 Department of Psychiatry, Universal college of Medical Science and Teaching Hospital, Bhairahawa, Nepal

**Keywords:** *cadavers*, *median nerve*, *variations*

## Abstract

**Introduction:**

Median nerve passes between two heads of pronator teres muscle while passing through the elbow. Detailed knowledge of these variations in the course of Median Nerve in relation to pronator teres and its neighboring structure is required for diagnosis of pronator syndrome. The aim of the study is to find out the proportion of Median Nerve passing below the ulnar head of pronator teres in cadavers of a medical college in Western Nepal.

**Methods:**

A descriptive cross-sectional study was carried out at the Department of Anatomy in a medical college of Nepal from 20^th^ July 2021 to 2^nd^ September 2021 after ethical clearance from the same institution (Reference number: UCMS/IRC/079/21). Variations in the course of the median nerve while passing through pronator teres were observed, recorded and photographed. Convenience sampling method was used. Data were analyzed using Microsoft Excel 2016. Point estimate at 95% Confidence Interval was calculated along with frequency and percentage.

**Results:**

Out of 54 prosected specimens of upper limbs, 4 (7.40%) (0.418-14.38 at 95% Confidence Interval) Median Nerve passed below the ulnar head of pronator teres muscle and in 50 (92.60%) specimens Median Nerve passed between two heads of pronator teres.

**Conclusions:**

Our study shows that the median nerve passed below the ulnar head of pronator teres muscle is higher as compared to other studies done in similar settings. Thus, knowledge of variations in the course of Median Nerve in elbow has immense importance in the academic and clinical arena.

## INTRODUCTION

Median nerve (MN) passes in the forearm between two heads of pronator teres (PT) muscle in elbow.^1^ Variations in course of median nerve in relation to pronator teres is frequently documented in surgical textbooks. One possible form of compression of the MN in the proximal part of the forearm is caused by variation in the humeral and ulnar heads of the Pronator Teres.^2^

Entrapment of the median nerve in the elbow and proximal forearm is one of the most common predisposing factors for pronator syndrome.^2^ Additionally, detailed knowledge of these variations in course of Median Nerve in relation to pronator teres and its neighboring structure is required for diagnosis of pronator syndrome, Median nerve electrostimulation, neuribiotization and open or minimally invasive median nerve surgery.^3,4^

This study aimed to find out the proportion of median nerve passing below the ulnar head of pronator teres in cadavers of a medical college in western Nepal.

## METHODS

A descriptive cross-sectional study design was conducted to observe variations in course of median nerve while passing through pronator teres muscle in human cadavers in the dissection hall of Department of Anatomy, Universal College of Medical Sciences Teaching Hospital, Bhairahawa, over the period of 20^th^ July 2021 to 2^nd^ September 2021. Ethical approval was taken from Institutional Review Committee of Universal college of Medical Sciences and Teaching Hospital, Bhairahawa, Rupandehi, Nepal (IRC UCMS, Ref: UCMS/IRC/079/21). Variations in the course of the median nerve while passing through pronator teres were observed, recorded and photographed. The sample size was calculated using the formula:

n = Z^2^ × (p × q) / e^2^

  = (1.96)^2^ × 0.02 × (1-0.02) / (0.05)^2^

  = 30.12

  = 31

Where,

n= required sample size
Z= 1.96 at 95% Confidence Interval (CI)
p= proportion of Median Nerve passing below the ulnar head of pronator teres in cadavers of a medical college in Western Nepal taken as 2% from previous study^5^
e= margin of error, 5% in this study

Hence, the sample size for the study was calculated and found to be 31. After addressing 10% for nonresponse, the final sample size calculated was 35. However, a sample size of 54 was taken.

However, a total fifty four formalin embalmed upper limb of cadavers of the department of anatomy were dissected exposing cubital fossa, pronator teres muscle and its relationship with median nerve. Convenient sampling method was used. The upper limb specimens who had any pathological lesions in limb region or any signs of trauma to limb were excluded for study. The specimens where sex were not determined were also excluded.

Variations in the course of the median nerve as it passes through the pronator teres muscle was observed, recorded and photographed. All the collected data were entered into Microsoft Excel 2016. Point estimate at 95% Confidence Interval was calculated along with frequency and percentage.

## RESULTS

Out of fifty four prosected specimens of upper limbs,

4 (7.40%) (0.418-14.38 at 95% Confidence Interval) median nerve passed below the ulnar head of pronator teres muscle (Table 1).

**Table 1:** Showing the course of median nerve while passing through pronator teres (n = 54).

Course	n (%)
Between Two heads of PT	50 (92.60)
Below Ulnar head of PT	4 (7.40)

Out of 54 prosected specimens, 8 (14.82%) were from female cadavers and 46 (85.18%) were from male cadavers (Table 2).

**Table 2:** Showing sex distribution of course of median nerve while passing through pronator teres muscle (n = 54).

Sex		Between two heads of PT n (%)	Below ulnar heads of PT n (%)
Male	46(85.18)	42 (91.30)	4 (7.40)
Female	8(14.82)	8(100)	0(0)

## DISCUSSION

Median nerve course while passing through pronator teres plays a pivotal role in diagnosing pronator teres syndrome. Median nerve passed between the two heads of the pronator teres muscle in most of the upper extremities (82%, 74%)^5,6^ and less often passed below the ulnar head (2%).^5^ Jamieson, et al. reported it passed through ulnar head in 12% specimens.^7^ In some it passed under both heads (12%).^6^

In our study median nerve passes between two heads of pronator teres in (92.60%) cases and in (7.40%) cases it passes below the ulnar head which is quite high compared to the other studies. A study conducted on Indian cadavers also had a similar result.^8^

In another study done in indian population by Basangauda, et al. the median nerve passed between the two heads of pronator teres muscle in (87.09%), deep to the pronator teres muscle in (4.83%), through the ulnar head of pronator teres muscle in (4.83%) and deep to the humeral head of pronator teres muscle.^9^

In a similar type of study done by Gaikwad, et al. noted thatmedian nerve passed between ulnar and humeral head in (92%) extremities.^10^

In contrast to this a study done by Olenwik, et al. in European population in cadavers MN passed between the heads of the PT muscle (74%) or under the muscle (26%).^6^ Christabel, et al. did a similar type of study where MN passed according to the norm between the two heads of the pronator teres muscle on most of the upper extremities (82%) and less often passed below the ulnar head (2%).^5^ In a similar type of study done by Caetano, et al. at Brazil both humeral and ulnar head was present in 86%. In 83.72%, the median nerve was positioned between the two heads of the pronator teres muscle; in 12.79%, it passed through the muscle belly of ulnar head of the PT muscle, and in 3.48%, posteriorly to both heads of the PT muscle.^11^

In a study done by Abe, et al. in fetus reported the absence of ulnar head in late stage fetuses and suggested possibly after birth - a small PT origin from the joint capsule appeared to obtain an aponeurosis connecting the muscle fiber to the ulna. This secondary change in PT morphology might explain the muscle variation seen in adults.^12^

In our study out of 8 (14.82%) upper limb were from female cadavers where all median nerve (100%) pass between two heads of pronator teres muscle and out of 46 (85.28%) specimens of upper limb from male cadaver 42 (91.30%) passes between two heads of pronator teres and 4 (7.40%) Median Nerve pass below ulnar head of pronator teres muscle. These results signal our attention towards gender differences in the course of the median nerve. Jhonson, et al. did a study over a period of 20 years and stated median nerve entrapment more in females as compared to males.^13^

Bae, et al. did an ultrasonographic study in 107 healthy Asians where they observed difference in Cross Sectional Area (CSA) of Median nerve in elbow region.^14^ Male had comparatively larger values as compared to female. They also stated an increase in cross sectional area of nerve with age. This was also supported by various other study.^15,16^

Farioli, et al. did a study in a larger population in relation to carpal tunnel syndrome and they suggested hormonal factors, anthropometric characteristics, and non-occupational exposure to biomechanical overload (e.g. household tasks) for more incidence of carpal tunnel syndrome in female as compared to males.^17^

Present study signal towards the variations in course of median nerve while passing through pronator teres below ulnar head heads is quite high as compared to other studies done in the European population. This finding was also supported by various other studies in the course of median nerve done in the Indian population.^9,10^ Ethnic difference may play a pivotal role for such variations.^18,19^ We further recommend others to conduct various other research on the general Nepalese population in this arena.

The limitations of the study is that the cadaver for our study was taken only from one medical college, where maximum of the cadaver was not fresh and embalmed more than three years ago, so there could have been formalin shrinkage.

## CONCLUSIONS

Median Nerve while passing through pronator teres has a wide range of variations. Prevalence of median nerve passing below the ulnar head of pronator teres was found high in our study as compared to other studies done in similar settings. It was also observed that this number was more in male cadavers as compared to females. This information could be helpful to establish a relationship between high incidences of pronator syndrome in male as compared to females. These variations help to diagnose various clinical conditions and surgical interventions. Clinicians should have ample knowledge about these variations.
